# Beyond bone effects: the role of denosumab in muscle Health – A systematic review

**DOI:** 10.1007/s40520-025-03285-0

**Published:** 2026-01-09

**Authors:** Chiara Ceolin, Chiara Ziliotto, Mario Virgilio Papa, Anna Bertocco, Giuseppe Sergi, Marina De Rui

**Affiliations:** 1 Geriatric Unit, Department of Medicine (DIMED), University of Padua, Padua, Italy; 2 Department of Neurobiology, Care Sciences and Society, Aging Research Center, Karolinska Institutet and Stockholm University, Stockholm, Sweden

**Keywords:** Denosumab, Sarcopenia, Falls, Muscle strength, Handgrip

## Abstract

**Background:**

Denosumab, a monoclonal antibody targeting RANKL, is widely used for the treatment of osteoporosis. In addition to its skeletal benefits, emerging evidence suggests that denosumab may also exert positive effects on muscle health by modulating inflammation, myostatin expression, and insulin sensitivity through the RANK/RANKL/OPG pathway.

**Aims:**

To systematically review the available literature on the effects of denosumab on muscle-related outcomes, including muscle strength, muscle mass, physical performance, and fall risk.

**Methods:**

A systematic review was conducted in accordance with PRISMA guidelines. Databases including PubMed, Embase, and Cochrane Library were searched through May 2025 for studies evaluating the impact of denosumab on muscle health in human subjects. Outcomes of interest included grip strength, lean muscle mass, gait speed, fall incidence, and physical performance tests.

**Results:**

Seven studies met the inclusion criteria, including randomized trials and observational cohorts. Most reported favorable outcomes for denosumab compared to bisphosphonates or placebo, particularly in grip strength and physical performance. Preclinical studies further support the biological role of RANKL in muscle dysfunction. However, one recent randomized controlled trial in older adults residing in long-term care settings found no significant effect on muscle outcomes, highlighting inconsistencies in the evidence.

**Discussion:**

Denosumab shows potential for improving muscle-related outcomes in older adults, particularly those with osteosarcopenia.

**Conclusions:**

Current evidence is heterogeneous and inconclusive. Further high-quality randomized trials are needed to clarify the effects of denosumab on muscle health and its possible role in sarcopenia prevention and management.

## Introduction

The aging process is often accompanied by musculoskeletal conditions that compromise physical autonomy and increase the risk of adverse outcomes. Among these, osteoporosis and sarcopenia stand out as prevalent and interrelated disorders that heighten susceptibility to falls, fractures, and disability in older adults [1]. Osteoporosis is marked by a decline in bone mineral density (BMD) and the deterioration of bone microarchitecture, leading to increased skeletal fragility [2, 3]. In parallel, sarcopenia involves a progressive reduction in muscle mass and strength, resulting in impaired mobility, balance, and functional independence [4]. The co-occurrence of these conditions—termed osteosarcopenia—presents a unique clinical challenge that requires treatment strategies capable of addressing both bone and muscle deterioration simultaneously [5, 6]. While pharmacological therapies such as bisphosphonates and denosumab are well established for osteoporosis, no approved medications currently exist for the treatment of sarcopenia [7].

To date, resistance exercise and sufficient dietary protein intake remain the primary strategies for managing sarcopenia, with non-pharmacological approaches continuing to represent the foundation of current clinical practice [8–11]. Nevertheless, increasing attention has been directed toward the possibility that certain antiresorptive drugs might exert beneficial effects on muscle tissue. In particular, denosumab, a monoclonal antibody that targets the RANK–RANKL interaction, has emerged as a candidate for dual-action therapy. While its ability to reduce bone resorption and prevent fractures is well-documented, growing evidence suggests that denosumab may also have positive effects on muscle strength and mass [12–14].

Due to the heterogeneity and limited consistency across existing studies, the exact role of denosumab in enhancing muscle-related outcomes remains unclear. Therefore, the present study aims to conduct a systematic review of the available evidence on the effects of denosumab on muscle health, with a focus on comparisons to other osteoporosis treatments regarding muscle strength, mass, physical performance, and fall risk.

## Materials and methods

### Data selection and collection process

This review adheres to the guidelines of the Preferred Reporting Items for Systematic Reviews and Meta-Analyses (PRISMA — http://www.prisma-statement.org/) [15]. To enhance the comprehensiveness of the review, the reference lists of included studies were also screened for additional relevant publications. The initial selection of articles was performed by a primary reviewer (C.Z.), and subsequently verified by a second independent reviewer (C.C.) to ensure consistency and accuracy in the inclusion process. Any discrepancies between reviewers were resolved through discussion, with input from a third reviewer (M.V.P.) when needed. The screening phase was facilitated using Rayyan, a collaborative web-based platform designed for managing systematic reviews.

To evaluate the methodological quality of the selected studies, two reviewers (C.Z. and C.C.) independently applied the Newcastle–Ottawa Scale (NOS) for observational and case-control studies, and the JADAD scale for the single clinical trial included. This review protocol was registered in the PROSPERO database (registration number: CRD420251001123). While a meta-analysis was initially planned, the substantial heterogeneity across studies—regarding study populations, control groups, outcome metrics, and data presentation—rendered quantitative synthesis infeasible and inappropriate.

### Sources of information

The Scopus, PubMed, and Cochrane databases were consulted from the initial publication date until May 2025.

### Search strategy

The databases were searched using the terms ‘denosumab,’ ‘anti-RANKL,’ ‘sarcopenia,’ ‘muscle health,’ ‘muscle strength,’ ‘muscle mass,’ ‘physical performance,’ ‘equilibrium,’ and ‘falls.’

### Eligibility criteria

The inclusion criteria were as follows: (a) patients with osteoporosis; (b) prescription of denosumab therapy for the treatment of osteoporosis; (c) comparison with other osteoporosis therapies; (d) outcomes: effects on balance, muscle strength, muscle mass, physical performance, falls; (e) studies published on the topic of interest using any study methodology, with a focus primarily on case-control, prospective, and cross-sectional studies; (f) availability of the full-text of the original research article. The exclusion criteria were: (a) abstracts, letters, and editorials; (b) studies not written in English.

### Data extraction

The titles and abstracts of the selected articles were examined to assess their relevance. The following data were extracted: (1) year, author, country; (2) study design; (3) sample size, including the number of female patients, mean/median age of participants; (4) type of population considered and number of participants; (5) dosage of the prescribed drug; (6) prescription of calcium and vitamin D supplementation or not; (7) tool for assessing muscle strength; (8) tool for assessing muscle mass/body composition; (9) tool for assessing balance/fall count assessment; (10) outcome on muscle mass/muscle strength/physical performance/balance/falls; and (11) main findings.

## Results

**Fig. 1:**
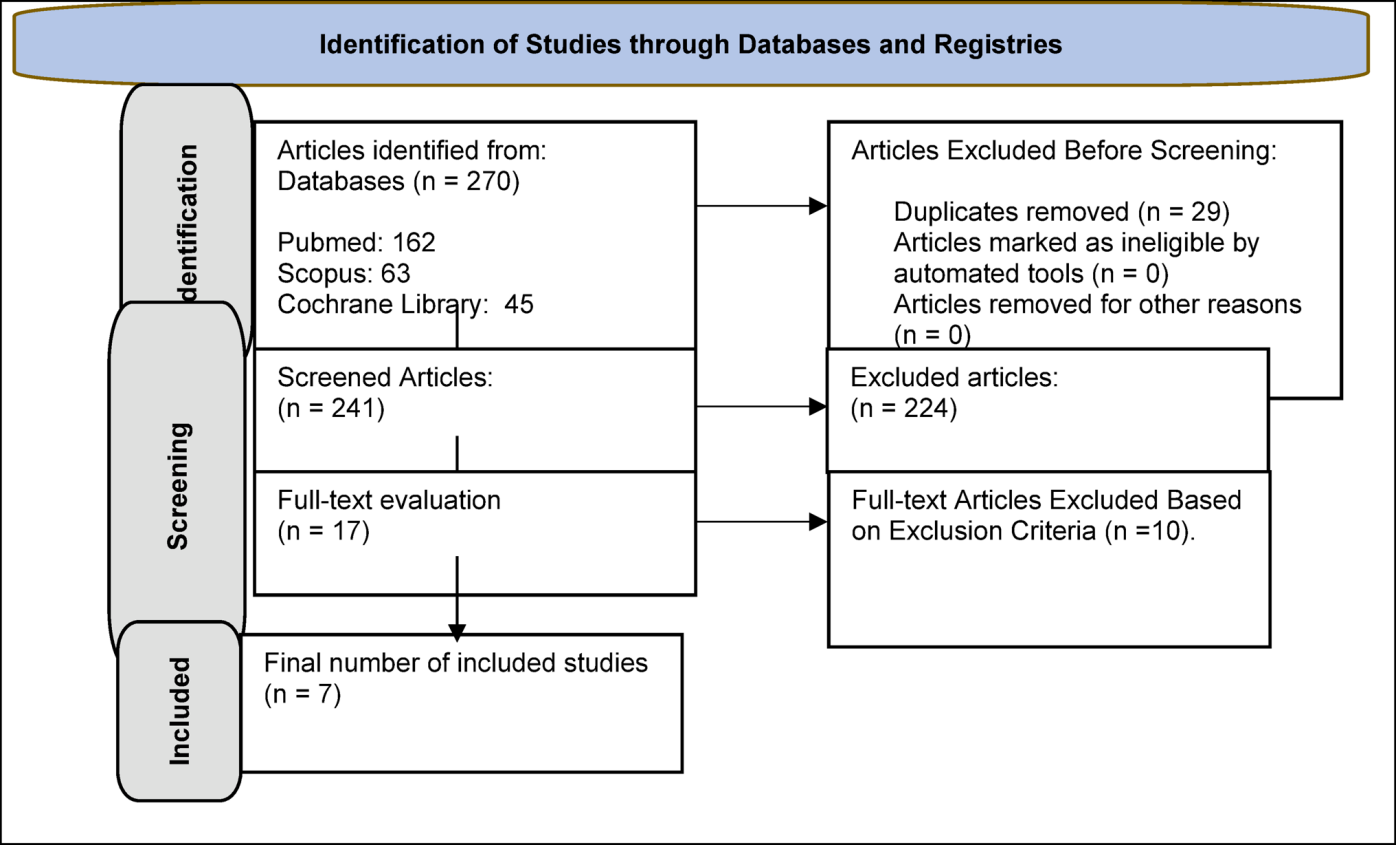
PRISMA Flow Diagram of the Study Selection Process with Reasons for Inclusion and Exclusion

A total of 270 studies were identified from the database searches, of which 29 duplicates were excluded. After reviewing the titles and abstracts, 224 articles were discarded, leaving 17 articles whose full manuscripts were examined in detail. An additional 10 articles were excluded as unsuitable, leaving 7 studies for full evaluation (Fig. 1). The NOS scores for the longitudinal studies and the JADAD score for the clinical trial of the included articles are reported in Table 1. After evaluation by two reviewers, the studies received an average NOS score of 5.5 and a JADAD score of 5 for the clinical trial, indicating good quality.

**Table 1:** NOS and JADAD scores assigned to the studies included in the review

Studio	NOS score	JADAD score
El Miedany, 2021	7	-
Bonnet, 2019	6	-
Phu, 2019	6	-
Pizzonia, 2021	4	-
Casabella, 2024	5	-
Rupp, 2022	5	-
Chotiyarnwong, 2019	-	5

Abbreviations: *NOS*: Newcastle-Ottawa Scale

Of the seven selected studies, three were prospective studies [12–14, 16], two were retrospective studies [17, 18], and one was a randomized controlled clinical trial [19]. The studies were conducted in Egypt [13], Switzerland [14], Thailandia, United Kingdom and Unites States of America [19], Italy [16, 18], Germany [17], and Australia [12].

The studies considered assessed parameters related to muscle mass and strength, physical performance tests, and fall risk in cohorts of patients receiving denosumab therapy, with the aim of identifying any differences compared to other prescribed osteoporosis therapies and/or placebo.

Table 2 presents the descriptive characteristics of the studies included in the systematic literature review; the results of the review are then reported, divided according to the different parameters considered: muscle mass, muscle strength, physical performance, balance, and fall risk.

**Table 2:** Descriptive characteristics of the studies included in the systematic literature review

Year, Authors, Country	Study design	Age (mean ± standard deviation), M:F	Population	No. Participants	Medication dosage (DmAB)	Supplementation with calcium/vitamin D	Controls	Muscle strength assessment	Muscle mass assessment	Falls evaluation	Findings
2021, El Miedany et al. Egypt	longitudinal multicenter controlled prospective study.	N.A., M: F = 303:104	Patients with postmenopausal/senile osteoporosis	DmAB (= 135), Control group (= 272)	60 mg sc injection every six months	All patients	Zol 5 mg IV injection (= 136), Aln 70 mg (= 136)	Hand-grip strenght (TUG, 4-m walk)	-	yes	In Dmab group (at 5 years of therapy) there was significant decrease in fall risk score, improvements in the grip strenght and gait speed. 1 year after stoppimg Dmab there is significant worsening of falls risk score, grip strenght; TUG and gait speed.
2019, Bonnet et al. Switzerland	Longitudinal multicenter, controlled, prospective study (GENEVA cohort)	DmAb 64.9 ± 1.5 yr control 65.7 ± 0.9 year, M:F = 0:40	Post-menopausal osteoporotic women	DmAB (= 20), Control group (= 20)	60 mg sc injection every six months	N.A.	Zol 5 mg IV injection (= 12), Aln 70 mg (= 8)	Handgrip strength	-	no	Only Dmab increased ALM and handgrip strenght, strogly correlated wth changes in lumbar spine BMD (not in the other groups)
2019, Chotiyarnwong Thailandia, UK, USA	Placebo-controlled trials	60–90 y (trial 1)	Post menopausal women with osteoporosis (trial 1) Post menopausal women with low bone mass (trial 2) women with non metastatic breast cancer receiving adjuvant aromatase inhibitors men with non methastatic prostate cancer receiving androgen deprivation therapy	DmAB (= 3886) Control group (= 3876)	60 mg sc injection every six months	Yes	Placebo		-	yes	Dmab can reduce the number of fallers by aproximately 20%. A greater riduction in falls was observedwith DmAB treatment in subjects < 75 years potentially reflecting the impact of other factors on fall risk in the oldest old.
2021, Pizzonia et al. Italy	Observational prospective study	> 65 y, M:F = 5:35	Old age patients with hip fracture due to low-energy trauma	98 enrolled at baseline 40 evaluated after 1 year (DmAb = 15), control group (= 26))	60 mg sc injection every six months	N.A.	Aln 70 mg	Handgrip strenght	-	no	A relatively early trend of osteoporosis improvement was observed in both BMD and bone microarchitecture (i.e., TBS) in the alendronate group compared to DmAB, based on a 1-year longitudinal observation. However, a trend of improvement in sarcopenia (RSMI) was noted in the DmAB group compared to the alendronate group. Additionally, handgrip measurements showed a positive longitudinal trend in both treatment groups (31 patients).
2024, Casabella et al. Italy	Retrospective cohort study	> 65 y, M:F = 0:30	60 patients (female sex) diagnosed with non metastatic breast cancer undergoing adiuvant endocrine therapy	DmAB (= 30), Control group (= 30)	60 mg sc injection every six months	N.A.	Aln 70 mg	N.A.	DXA	no	Signifcant improvements in TBS at the lumbar spine, RSMI and whole-body composition (arms, legs, and trunk) were observed in the DmAB group compared with the BF group.
2022, Rupp et al. Germany	Retrospective cohort study	> 65 y, M:F 8:52 for DmAB, 4:26 for alendronate	Osteopenic or osteoporotic patients	DmAB (= 60), Control group (= 90)	60 mg sc injection every six months	N.A.	Bp (= 30), Vit D suppl alone (= 60)	Hand-grip strenght chair rising test force and time	DXA	no	Dmab group showed a significantly higher increase in chair rising test force compared to BP group. Neither the changes in BMD nor in bone metabolic parameters were associated with chjanges in muscle performance
2019, Phu et al. Australia	Longitudinal study	> 65 y, M:F 8:23 for DmAb, 10:18 for Zol	Community-dwelling older adults with history or risk of falls and/or fractures evaluated at time 0 and after 6 months.	DmAB (51), Control group (= 28)	60 mg sc injection every six months	Yes	Zol 5 mg IV injection	Hand-grip strength, SPPB (standing balance, 4 m gait speed, 5-time sit to stand test, TUG and FSST)	-	yes (refered n of falls) Balance Rehabilitation Unit (LoS, center of pressure and sway velocities) FES-I and ABC (activities specific balance confidence scale)	At follow up, Dmab improved gait speed, TUG and FSST. Fear of falling reduced in FES-I and impreved in ABC. trend toward improvements was found in SPPB score and LOS. Zol improved gait speed and TUG. Compared to Zol Dmab showed lower FSST and FES-I at six months. No differences in fal or fractures.

*Abbreviations*: DmAb: Denosumab; Zol: Zoledronate; Aln: Alendronate; TUG: Timed Up and Go; DXA: Dual-energy X-ray Absorptiometry; BMI: Body Mass Index; BMD-LS: Bone Mineral Density at the Lumbar Spine: ALM: Appendicular Lean Mass; RSMI: Relative Skeletal Muscle Index; TBS: Trabecular Bone Score; Bp: Bisphosphonates; FSST: Four Square Step Test; FES-I: Falls Efficacy Scale-International

### Muscle Mas

Casabella et al. conducted a body composition analysis involving a cohort of sixty women diagnosed with non-metastatic breast cancer, all receiving adjuvant therapy with either denosumab or alendronate [18]. The study demonstrated notable enhancements in muscle-related parameters among participants treated with denosumab, including significant increases in lean body mass (OR = 2.054, *p* = 0.0021) and total fat-free mass (OR = 2.049, *p* = 0.0022). Moreover, a statistically significant upward trend was identified in the Relative Skeletal Muscle Index (OR = 0.418, *p* = 0.0392) after one year of treatment, favoring the denosumab group over those receiving alendronate [17].

### Muscle strength

Multiple studies included in this review evaluated muscle strength through handgrip dynamometry. In a large multicenter, longitudinal prospective study, El Miedany et al. assessed 407 postmenopausal women with osteoporosis and reported a significant gain in grip strength after five years in the subgroup treated with denosumab (*n* = 135), with an average increase of + 4.3 kg (*p* = 0.01). This effect was notably greater than in the control group (*n* = 272), which received either oral or intravenous bisphosphonates [13].

Similarly, another multicenter prospective study compared 20 patients undergoing long-term denosumab treatment (mean duration: 3 years) with 20 patients on bisphosphonates. Results showed that denosumab not only enhanced Appendicular Skeletal Muscle Mass Index (ASMMI) but also significantly increased handgrip strength (0.66 ± 2.2 kg and 3.22 ± 10.0 kg, respectively). In contrast, those receiving bisphosphonates showed minimal changes (− 0.06 ± 0.39 kg and − 0.07 ± 6.6 kg), while untreated participants experienced a decline (− 0.36 ± 1.03 kg and − 1.39 ± 2.4 kg). All changes were statistically significant (*p* < 0.05) [14]. Notably, improvements in both ASMMI and grip strength were strongly correlated with increases in lumbar spine BMD (r² = 0.82 and 0.81, respectively; *p* < 0.001) among denosumab-treated individuals, a pattern not observed in the other groups.

A retrospective analysis by Rupp et al. also reported a positive annual percentage change in grip strength in a cohort of 60 osteoporotic patients treated with denosumab [17]. Although patients receiving bisphosphonates (*n* = 30) also experienced modest gains, the magnitude was smaller compared to denosumab (+ 0.78% ± 8.23% vs. +5.14% ± 25.49%). In contrast, the control group receiving only vitamin D (*n* = 60) exhibited a decline in grip strength (− 6.05% ± 10.22%) [17].

Further evidence comes from a study involving older women recovering from fragility hip fractures. After one year of follow-up, improvements in handgrip strength were recorded in both treatment arms: patients receiving alendronate (19 of 22) improved by an average of + 0.85 kg (SD = 4.8), while those treated with denosumab (12 of 13) showed a slightly higher mean increase of + 0.97 kg (SD = 6.0), indicating a favorable trajectory over time [16].

### Physical performance

Several of the included studies evaluated physical performance using standardized functional tests. In the longitudinal study conducted by El Miedany et al., individuals treated with zoledronate or alendronate exhibited modest but statistically significant improvements in the Timed Up and Go (TUG) test (reductions of 0.7 and 0.8 s, respectively; *p* = 0.05) and in gait speed (both groups: +0.07 m/s; *p* = 0.05). However, the denosumab-treated cohort demonstrated more pronounced gains over the five-year treatment period, with TUG time improving by 1.5 s (95% CI: −2.2 to 0.1; *p* = 0.001) and gait speed increasing by 0.1 m/s (95% CI: 0.03–0.2; *p* = 0.001) [13].

Additional support for the benefits of denosumab on functional outcomes comes from a retrospective cohort study by Rupp et al., in which patients receiving denosumab showed greater enhancement in the Chair Rise Test (CRT) performance compared to those on bisphosphonates. Specifically, the denosumab group exhibited an average annual percentage improvement in CRT strength of + 8.20% ± 14.38%, exceeding the gains observed in the baseline group (+ 5.82% ± 12.74%) and the bisphosphonate group (+ 0.95% ± 8.61%), though the latter two showed no significant difference between them [17].

Moreover, a separate investigation involving community-dwelling older adults with prior falls or high fracture risk found that six months of denosumab therapy led to significant improvements in multiple physical performance measures. Patients receiving denosumab showed superior functional outcomes compared to those treated with zoledronate, including a notable increase in walking speed (+ 0.06 m/s; 95% CI: 0–0.1; *p* = 0.041), a shorter Timed Up and Go (TUG) duration (− 1.7 s; 95% CI: −3.4 to − 0.1; *p* = 0.041), and improved performance on the Four Square Step Test (− 1.7 s; 95% CI: −2.7 to − 0.6; *p* = 0.003) [12].

### Risk of falls

The final domain explored in this review concerns balance and the risk of falls. A pooled analysis of five randomized, placebo-controlled trials investigating denosumab revealed that administering the drug subcutaneously every six months was associated with an approximately 20% reduction in fall incidence [19]. Among participants, 5.8% of those receiving placebo (289 falls in 5006 individuals) reported at least one fall, compared to 4.6% in the denosumab group (231 falls in 5030 individuals). When adjusted for exposure time, the fall incidence rate was 2.3 in the placebo group and 1.8 in the denosumab group, yielding a rate ratio of 0.77 (95% CI: 0.66–0.90) [19]. Excluding fracture-related falls, the adjusted rates were 2.0 (placebo) versus 1.5 (denosumab), with an identical rate ratio of 0.77 (95% CI: 0.65–0.91). Across all studies included in the pooled analysis, the proportion of participants experiencing at least one fall was either lower or similar in the denosumab arms compared to placebo [19].

Further analysis using Cox proportional hazards models showed hazard ratios ranging from 0.16 (95% CI: 0.02–1.37) to 0.99 (95% CI: 0.14–7.04), with a pooled hazard ratio (HR) of 0.79 (95% CI: 0.66–0.93), indicating a 21% overall risk reduction [19]. Similarly, Kaplan–Meier estimates reported a fall incidence of 6.5% in placebo recipients versus 5.2% in those treated with denosumab, again with an HR of 0.79 (95% CI: 0.66–0.93; *p* = 0.0061) [19]. Interestingly, subgroup analysis revealed that the reduction in fall risk was more pronounced among participants under 75 years of age (interaction *p* = 0.012), where denosumab led to a 35% reduction in risk (HR = 0.65, 95% CI: 0.52–0.82). No significant effect was observed in those aged 75 and older (HR = 1.01, 95% CI: 0.78–1.31), though a possible early benefit could not be ruled out [19].

Complementary findings were reported by El Miedany et al., who observed a significant decline in fall risk after five years of denosumab treatment (mean change: −1.9; 95% CI: −2.8 to − 0.7; *p* = 0.001), an effect not replicated in the bisphosphonate groups (oral and intravenous, *p* = 0.18 and 0.25, respectively) [13]. Importantly, following discontinuation of denosumab, participants experienced significant worsening in fall risk, handgrip strength, TUG performance, and walking speed (*p* = 0.001). Such deteriorations were not observed after cessation of alendronate or zoledronate [13].

In a separate longitudinal study assessing self-perceived fall risk, patients reported improved scores on the Falls Efficacy Scale International (FES-I), with a reduction of 3.1 points (95% CI: −5.5 to − 0.8; *p* = 0.01), and higher confidence as reflected in a 7.8% increase on the Activities-Specific Balance Confidence scale (95% CI: 1.0–14.6; *p* = 0.025) [12]. Additionally, these individuals showed a trend toward functional improvement, with borderline significance on the Short Physical Performance Battery (SPPB) score (+ 1.1 points; 95% CI: −0.04 to 2.3; *p* = 0.058) and enhanced limits of stability (+ 11.9 cm²; 95% CI: −0.4 to 24.3; *p* = 0.058) [12].

## Discussion

This systematic review examined the potential role of denosumab in influencing muscle-related outcomes, including muscle strength, muscle mass, physical performance, and fall risk. Although a number of studies reported beneficial effects, the substantial heterogeneity in study populations, comparators, and outcome assessment methods limits the ability to draw firm conclusions. As such, the current body of evidence should be considered exploratory and primarily hypothesis-generating.

**Fig. 2:**
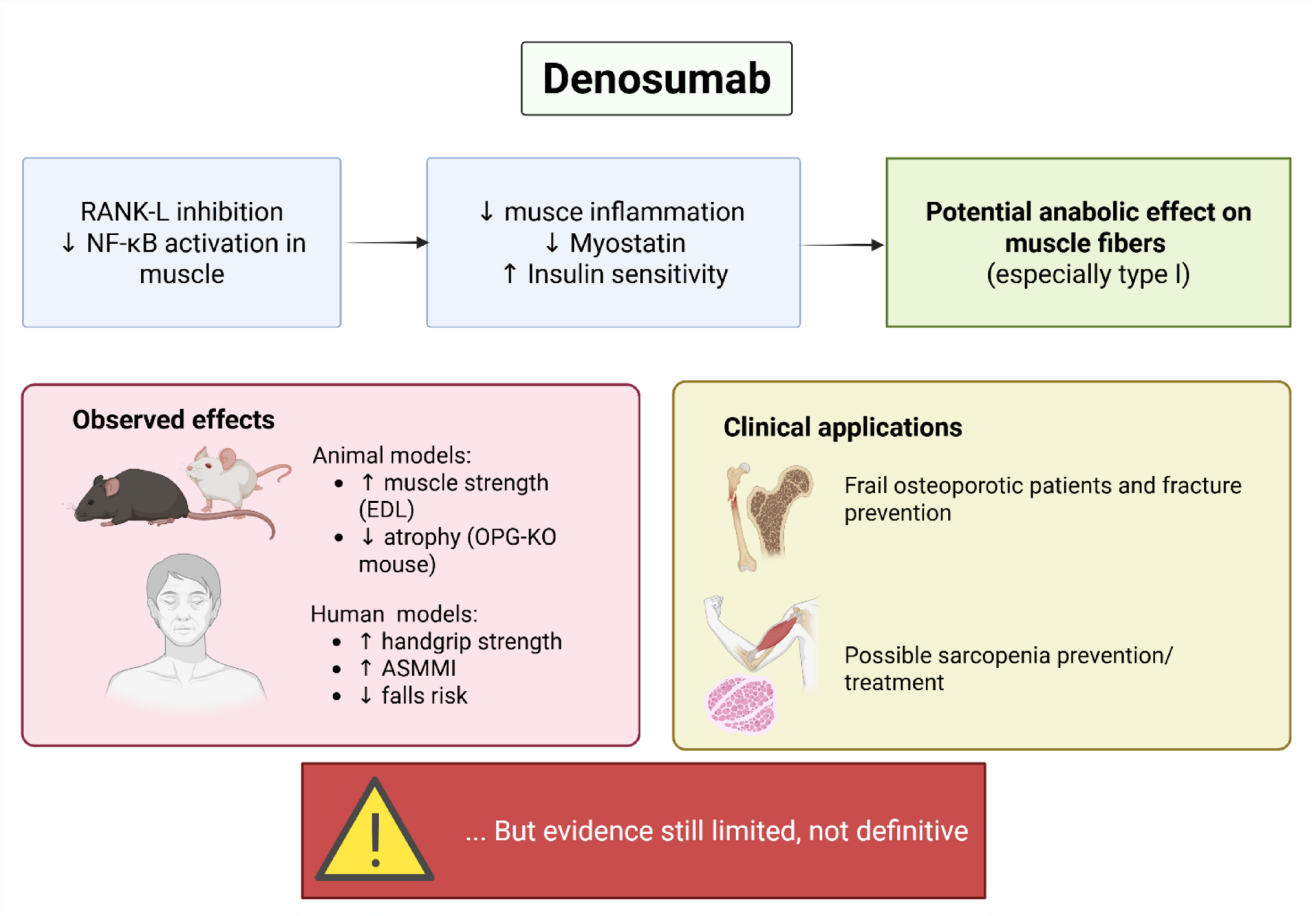
Mechanistic and Clinical Evidence Linking Denosumab to Muscle Function: A Conceptual Overview

Osteosarcopenia, characterized by the co-occurrence of osteoporosis and sarcopenia, is increasingly acknowledged as a clinically relevant condition, particularly in older individuals, due to its strong association with elevated risks of falls, fractures, functional impairment, and mortality [20, 21]. Within this framework, pharmacological agents that can simultaneously address bone loss and muscle deterioration hold significant therapeutic promise. One such agent is denosumab, a monoclonal antibody targeting RANKL, which is well established as an anti-resorptive treatment for osteoporosis. Emerging evidence suggests that the RANK/RANKL/OPG signaling pathway, beyond its role in bone metabolism, may also be involved in muscle homeostasis. Specifically, components of this pathway have been implicated in modulating inflammatory responses, myostatin expression, and insulin sensitivity within skeletal muscle tissue [21, 22]. Preclinical studies reinforce this idea: in murine models, elevated RANK and RANKL expression has been observed in oxidative muscle fibers—particularly in the soleus—compared to other muscle types and soft tissues such as the liver, adipose tissue, and intestines [14]. In transgenic mice overexpressing human RANKL (huRANKL), researchers observed reduced muscle mass and strength, diminished glucose uptake, and elevated levels of pro-inflammatory and anti-myogenic markers including myostatin and Ptp-RG. These physiological disruptions were accompanied by increased insulin resistance, mediated through NF-κB pathway activation [14]. Remarkably, pharmacological blockade of RANKL—via OPG-Fc or denosumab—mitigated these adverse effects in both huRANKL and Pparb⁻/⁻ mouse models of osteosarcopenia. These findings suggest a potentially independent role for the RANK/RANKL axis in driving muscle dysfunction, regardless of the initiating condition [14]. A visual representation of these mechanisms is provided in Fig. 2, which outlines the proposed biological actions of denosumab on skeletal muscle, supported by both animal and clinical data, and highlights its prospective therapeutic relevance beyond bone health.

In clinical research, the phase 3 FREEDOM trial demonstrated that denosumab significantly enhances BMD at key skeletal sites, including the femur, lumbar spine, and distal radius, and effectively reduces the incidence of vertebral, non-vertebral, and hip fractures in postmenopausal women diagnosed with osteoporosis [23]. In addition to its established skeletal benefits, the trial also evaluated fall incidence, revealing a significantly lower rate of falls among participants receiving denosumab compared to those on placebo [23]. These findings suggest that inhibiting RANKL may provide advantages beyond bone protection—potentially improving muscle performance and functional stability, thereby mitigating the risk of falls and other adverse outcomes.

Additional insights into the broader role of the RANK/RANKL/OPG pathway in musculoskeletal physiology emerge from preclinical investigations. For example, Pin et al. (2022) reported that elevated systemic RANKL levels were linked to musculoskeletal deterioration in both animal models and human subjects with non-metastatic ovarian cancer and cachexia, pointing to a tumor-driven mechanism contributing to both bone and muscle degradation—even in the absence of metastases [24]. Similarly, Hamoudi et al. (2020) demonstrated that mice genetically deficient in osteoprotegerin (OPG) not only developed osteoporosis but also exhibited atrophy of fast-twitch muscle fibers, reduced strength, and impaired mobility. These deficits were partially reversed through anti-RANKL therapy [25, 26]. Building on these observations, researchers have also explored the application of RANKL blockade in degenerative muscle disorders. In Duchenne muscular dystrophy models, Dufresne et al. found that full-length OPG-Fc administration enhanced extensor digitorum longus muscle strength, improved resistance to contraction-induced damage, and increased treadmill endurance. Interestingly, these effects surpassed those observed with anti-RANKL or anti-TRAIL monotherapies [27]. Supportive evidence from clinical observation includes a case report involving a 66-year-old male with facioscapulohumeral muscular dystrophy who transitioned from teriparatide to denosumab after two years of osteoporosis management. Following the initial injection of denosumab, the patient reported a marked short-term improvement in muscular symptoms, which recurred after subsequent doses administered every 40–42 days. This clinical response was corroborated by better performance on grip strength assessments and functional mobility tests such as the TUG and Sit-to-Stand evaluations [28].

Our systematic review reinforces the potential role of denosumab in improving muscle health, particularly in comparison to oral bisphosphonate therapy. The evidence suggests a positive impact on grip strength, muscle mass, functional performance, and fall prevention. However, the available data remains inconsistent. A recent double-blind, placebo-controlled randomized trial involving older adults living in long-term care facilities found no statistically significant differences between denosumab and placebo over a 24-month period with respect to lean muscle mass, gait speed, or overall physical performance. Nonetheless, there were non-significant trends suggesting potential benefits of denosumab among female participants, particularly in terms of grip strength and chair stand test performance [29]. In the Italian clinical context, denosumab is authorized as a second-line treatment, offering a valuable alternative for individuals who are intolerant to bisphosphonates or have comorbid conditions such as chronic kidney disease [30]. In such frail populations, a pharmacological agent capable of addressing both bone fragility and muscle decline may deliver meaningful therapeutic advantages. Improving muscle strength and function in these individuals could encourage greater physical activity, reduce fall and fracture risk, shorten hospital stays, and help preserve functional independence and quality of life. An additional benefit of denosumab lies in its user-friendly dosing regimen: a single subcutaneous injection every six months, which stands in contrast to the more frequent and often poorly tolerated administration of bisphosphonates, particularly due to gastrointestinal side effects [31]. This simplified delivery method may enhance treatment adherence in older adults and those with complex health profiles.

It is important to acknowledge, however, that previous attempts to synthesize the literature on this topic have encountered challenges. A recent meta-analysis was retracted due to concerns about data validity and flawed methodology [32]. In conducting our review, we identified several critical issues in the existing literature: inclusion of pooled data analyses, and outcome reporting in inconsistent units (e.g., kilograms, percentages, T-scores). Given these limitations, and in order to avoid introducing further bias, we deliberately chose not to perform a quantitative meta-analysis. Instead, we presented a structured qualitative synthesis of the literature, which we believe more accurately reflects the current state of knowledge and the need for further high-quality research.

### Limits and Strengths

Several limitations of this review must be acknowledged. First, the number of eligible studies was limited, and most were observational in nature. Second, considerable heterogeneity in clinical design, populations, interventions, and outcome measures precluded meta-analytic pooling. Third, some included studies involved non-osteoporotic or oncologic populations, thereby limiting the generalizability of findings. Finally, only English-language studies were considered, potentially excluding relevant data from other contexts. Nevertheless, this review was conducted in accordance with PRISMA guidelines and is, to our knowledge, one of the few to critically assess the relationship between denosumab and muscle-related outcomes. Its main strength lies in the comprehensive and rigorous evaluation of an emerging area of research with significant clinical implications, especially for older adults. The growing attention to the interplay between bone and muscle health underscores the relevance of this topic and highlights the need for integrated therapeutic approaches in frail individuals and those with multimorbidity.

## Conclusions

Denosumab may offer ancillary benefits on muscle function in addition to its established role in bone health, but current evidence remains inconclusive. Although preclinical studies provide biological plausibility, and early clinical observations are encouraging, robust randomized controlled trials with standardized endpoints are urgently needed.

Future research should aim to clarify the role of the RANKL pathway in muscle regulation and assess the clinical effectiveness of denosumab in preventing or treating sarcopenia, particularly in frail, elderly populations. Until then, any extrapolation should be made cautiously, and clinical decisions should continue to be guided primarily by approved indications and high-quality evidence, though emerging data may soon warrant dedicated therapeutic studies in sarcopenic populations.

## Data Availability

No datasets were generated or analysed during the current study.
